# Antibiotic resistance, biofilm formation, and virulence factors of isolates of *staphylococcus pseudintermedius* from healthy dogs and dogs with keratitis

**DOI:** 10.3389/fvets.2022.903633

**Published:** 2022-08-10

**Authors:** Zhihao Wang, Long Guo, Jun Li, Jianji Li, Luying Cui, Junsheng Dong, Xia Meng, Chen Qian, Heng Wang

**Affiliations:** ^1^College of Veterinary Medicine, Jiangsu Co-Innovation Center for Prevention and Control of Important Animal Infectious Diseases and Zoonoses, Yangzhou University, Yangzhou, China; ^2^Joint International Research Laboratory of Agriculture and Agri-Product Safety of the Ministry of Education, Yangzhou, China

**Keywords:** *staphylococcus pseudintermedius*, cornea ulcer, antibiotic-resistance, virulence factors, canine

## Abstract

Canine bacterial keratitis is a common infection that can potentially threaten vision. *Staphylococcus pseudintermedius* (*S. pseudintermedius*) is an opportunistic pathogen that has been isolated from the canine conjunctival sac but there are only a few reports on the role of this bacterium in canine keratitis. This study focused on the distribution rate of *S. pseudintermedius* in the canine conjunctival sac, and the antibiotic resistance, biofilm-producing ability, and dissemination of virulence factors in strains of *S. pseudintermedius* isolated from healthy dogs and dogs with keratitis. The study included 35 healthy dogs and 40 dogs with keratitis. Bacterial species were confirmed by matrix-assisted laser desorption ionization-time of flight mass spectrometry (MALDI–TOF MS). Strains of *S. pseudintermedius* were screened for resistance against nine different antibiotics by the Kirby–Bauer assay. The ability to produce biofilm was investigated by microtiter plate assay (MtP) and amplification of *icaA* and *icaD* genes. Virulence factors in the strains were also evaluated. A total of 132 aerobic bacteria were isolated from the 119 samples in the study. Among them, 67 bacterial strains were isolated from 70 eyes of healthy dogs, and 65 bacterial strains were isolated from 49 eyes of dogs with keratitis. The prevalence of *S. pseudintermedius*, which was the most frequent bacterial isolate in both the groups, was 20.9% in the healthy group and 23.08% in the keratitis group. Most of the isolates of *S. pseudintermedius* were sensitive to rifampin (96.6%), oxacillin (100%), and neomycin (96.6%), and resistant to tetracycline (96.6%). Virulence factors such as *lip* (96.6%), *hlgB* (96.6%), and *hlgA* (96.6%) were found in most of the isolates, and 89.66% of isolates were classed as biofilm producers. In conclusion, *S. pseudintermedius* was the common bacterium in the conjunctivital sac of the healthy dogs and dogs with keratitis in Yangzhou, China, and the presence of virulence factors and biofilm-formation ability were high in the strains isolated from the dogs with keratitis.

## Introduction

The cornea is part of the eye exposed to the outer environment and thus most likely to sustain damage due to various insults (1). The eye resists a constant invasion of potentially pathogenic microorganisms but often falls victim to certain conditions, such as systemic immune disease or corneal damage (2–5). Bacterial keratitis in dogs is a common disease that has a short course, poor prognosis, and treatment difficulties, and can potentially threaten vision in the animals.

In general, the ocular flora environment is varied and complex. Understanding the primary pathogens of bacterial keratitis in this area and their antibiotic resistance is crucial for the selection of drugs during the clinical disease. The incidence of keratitis was reported to be related to the breed, age, and region of the dog (6). Although the surveys were independent and conducted in different geographic locations, Gram-positive organisms predominated, and *Staphylococcus* spp. were often identified (2).

*Staphylococcus pseudintermedius* (*S. pseudintermedius*) is a component of the *Staphylococcus intermedius group* (SIG), which also includes *S. intermedius* and *S. delphini* (7). As a zoonotic pathogen and opportunistic bacterium, *S. pseudintermedius* is most frequently associated with animal diseases such as pyoderma, otitis externa, and keratoconjunctivitis (6, 8–11). Human infections with *S. pseudintermedius* are less common than those in dogs, but reports on the identification of *S. pseudintermedius* as a human pathogen are increasing (12, 13). There is some evidence that *S. pseudintermedius* is the most common bacteria inducing corneal ulcers in animals. In a survey from 2005 to 2007 in Korea, 67 *Staphylococcus* strains were isolated from the conjunctival sac of 33 dogs with keratitis, of which 50 strains were *S. pseudintermedius*, thus, the isolation rate was 83.6% (4). A survey from 2009 to 2013 in Switzerland compared and analyzed the types of pathogenic bacteria-causing corneal ulcers in dogs, cats, and horses. The isolation rate for *S. pseudintermedius* was higher in dogs (9.7%) compared with that in cats (9.4%) and horses (2.9%) (4). Furthermore, in 2016, a survey of 56 dogs with bacterial ulcerative keratitis showed that the isolation rate for *S. pseudintermedius* was 25.2% (14), while a survey from 2014 to 2020 in 476 dogs presenting with suspected bacterial keratitis indicated that 26.7% of infections were caused by *S. pseudintermedius* (15).

Without using matrix-assisted laser desorption/ionization time-of-flight (MALDI–TOF), it can be difficult to differentiate between *S. pseudintermedius* and *S. intermedius* (16). *S. pseudintermedius* was first recognized and described in 2005 (17), followed by the first human cases reported in 2006 (18), and the first molecular identification protocol was published in 2009 (17). The epidemiology, pathogenicity, and resistance of *S. pseudintermedius* have received increasing attention. A literature review from 1980 to 2013 indicated that *S. pseudintermedius* has a higher level of resistance to amikacin, gentamicin, and enrofloxacin in recent years (16). A survey of antibiotic resistance and molecular characterization for 50 strains of *S. pseudintermedius* isolated from canines with keratitis showed that the strains were the most susceptible to amoxicillin/clavulanic acid (94%) and highly resistant to tetracycline (94%) and penicillin (92%) (18). In total, sixteen (88.9%) mecA-positive strains of *S. pseudintermedius* were also resistant to oxacillin, tetracycline, and penicillin (18). *S. pseudintermedius* carries many virulence factors, such as elastin-blinding protein (encoded by the gene *ebpS*), exfoliative toxin SIET (*siet*), and lipase (*lip*), which facilitate adhesion of *S. pseudintermedius* to the host extracellular matrix components, damaging host cells and impeding the immune system (10). *S. pseudintermedius* has developed antimicrobial resistance because of the selective pressures from the indiscriminate use of antimicrobial agents, and multidrug-resistant (MDR) strains have emerged, namely, methicillin-resistant *S. pseudintermedius* (13, 19). Furthermore, biofilm formation can facilitate the survival of *S. pseudintermedius* under the pressure of antimicrobial agents and help the bacterium evade the host immune response, which can give rise to persistent infection (20).

The objective of this study was to provide information on *S. pseudintermedius* isolates from the eyes of dogs with or without keratitis, and assess the ability of biofilm formation, antibiotic resistance, and the distribution of the virulence genes of the isolates.

## Materials and methods

### Animals

Ocular specimens were collected from 75 dogs, comprising 35 dogs that were proved healthy without keratitis and 40 dogs that were taken for veterinary consultation regarding the keratitis. A total of 119 samples were collected from December 2020 to December 2021. All the healthy dogs did not have any external ocular disease by routine ophthalmic examination (namely, Schirmer tear test, slit-lamp examination, and fluorescein stain strips). From the 49 samples obtained from 40 dogs with keratitis, 9 dogs had a binocular disease, and 31 dogs had a monocular disease. The clinical symptoms of keratitis in the dogs included ocular pain, photophobia, lacrimation, eyelid closure, corneal cloudy ulcer or perforation, and positive fluorescein sodium staining.

### Specimen collection

For collecting the samples, the examiner wore sterile gloves. The dog was placed on the examination table and the head was held. To avoid contamination from eyelid and eyelash, the upper and lower eyelids were wiped with an iodophor cotton ball before sample collection. The eyelids were then opened, and the eyes were flushed with physiological saline solution. A sterile swab was moistened and then rotated counterclockwise over the cornea (also including the ulcer) and conjunctival sac to collect samples. The swab was placed into a sterile tube immediately and transported to the laboratory.

### Isolation and identification of *S. pseudintermedius*

Each swab was inoculated into 5% sheep blood agar, Chapman Agar, and MacConkey agar. All the agars were incubated at 37°C in an aerobic environment for 48 h. Based on the colonial morphology, catalase testing, and Gram-staining, single colonies were selected for purification culture. Samples with no bacterial growth for more than 48 h were judged negative. Isolated organisms initially identified by morphology and biochemical methods (colony morphology, Gram-staining, and catalase testing), were then identified by MALDI–TOF (MALDI Biotyper, Bruker Daltonics, Germany) (21–25).

### Antimicrobial susceptibility testing

Antimicrobial susceptibility tests (ASTs) of *S. pseudintermedius* were determined by the agar disk diffusion method according to the guidelines of the Clinical Laboratory Standards Institute (CLSI) (26). Briefly, bacteria were inoculated on Mueller–Hinton agar at 35 ± 1°C in an aerobic chamber, and the zone diameter of resistance was determined after 18 h. *Staphylococcus aureus* ATCC 29213 was used for quality control. Antimicrobial agents often used clinically to treat corneal ulceration that were selected for use in the AST, namely, tetracycline (TE, 30 μg), chloramphenicol (C, 30 μg), rifampicin (RA, 5 μg), tobramycin (TO, 10 μg), neomycin (N, 30 μg), ciprofloxacin (CIP, 5 μg), erythromycin (E, 15 μg), levofloxacin (LVX, 5 μg), and oxacillin (OX, 1 μg). All the antimicrobial agents were purchased from the Hangzhou Microbial Reagent Co., Ltd.

### DNA extraction and primer typing

The Luria–Bertani (LB) medium was inoculated with a single bacterial colony and incubated at 37°C overnight. Next, a 1.5 ml culture medium was centrifuged at 12,000 rpm for 1 min. Bacterial DNA was subsequently extracted using the TIANamp Bacteria DNA kit (TIANGEN) according to the manufacturer's instructions.

Primers and protocols available in Kmieciak et al. (10), Futagawa-Saito et al. (27), Casagrande Proietti et al. (28), Lautz et al. (29), Lovseth et al. (30), Lina et al. (31) were adopted to amplify the genes *ebpS, spsE, lukS-I, lukF-I, lip, hlgA, hlgB, PVL, tsst, siet, icaA*, and *icaD* (Table 1). Primers were targeted to the regions of unique sequence within each gene.

**Table 1:** Primers, amplicon size, sequence, and annealing temperature.

**Gene**	**References**	**Protein/Toxin**	**Primer**	**Sequence (5^′^-3^′^)**	**Size of amplicons**	**Annealing temperature (°C)**
*ebpS*	(10)	Elastin-blinding protein	*ebpS-F*	AGACGCCACAGAAAAAGA	1040bp	54
			*ebpS-R*	GCAGATTGACCTTGTTGA		
*spsE*	(10)	Fibrinogen-/ fibronectin-blinding protein	*spsE-F*	TTTCTCGTTTCTGGGCGT	1600bp	54
			*spsE-R*	GCGTCTTCTGGTTATCGT		
*lip*	(10)	Lipase	*lip-F*	GGAAAAGCAGCAGAAAGAA	1601bp	54
			*lip-R*	GGGTGCTGTAGAAATA		
*hlgA*	(10)	γ-Hemolysin A component	*hlgA-F*	CTTCTTCCACTTATTACACC	718bp	56
			*hlgA-R*	CACTTGTATCGTTTATC		
*hlgB*	(10)	γ-Hemolysin B component	*hlgB-F*	GGGGGGCTAAGTAAGTAATGT	475bp	56
			*hlgB-R*	GCGCCATTTGGTTTATGT		
*lukF-I*	(27)	Leukotoxin F component	*lukF-F*	CCTGTCTATGCCGCTAATCCA	572bp	57
			*lukF-R*	AGGTCATGGAAGCTATCTCGA		
*lukS-I*	(27)	Leukotoxin S component	*lukS-F*	TGTAAGCAGCAGAAAATGGGG	503bp	57
			*lukS-R*	GCCCGATAGGACTTCTTACAA		
*icaA*	(28)	ica locus A	*icaA-F*	ACTGTTTCGGGGACAAGCAT	134bp	60
			*icaA-R*	ATTGAGGCTGTAGGGCGTTG		
*icaD*	(28)	ica locus D	*icaD-F*	CGTTAATGCCTTCTTTCTTATTGCG	166bp	56
			*icaD-R*	ATTAGCGCACATTCGGTGTT		
*siet*	(29)	exfoliative toxin SIET	*siet-F*	ATGGAAAATTTAGCGGCATCTGG	359bp	56
			*siet-R*	CCATTACTTTTCGCTTGTTGTGC		
*tsst*	(30)	toxic shock syndrome toxin	*tsst-F*	GCTTGCGACAACTGCTACAG	559bp	55
			*tsst-R*	TGGATCCGTCATTCATTGTTAT		
*PVL*	(31)	Panton- Valentine leucocidin	*PVL-F*	ATCATTAGGTAAAATGTCTGGACAT GATCCA	432bp	55
			*PVL-R*	GCATCAATGTATTGGATAGCAAAAGC		

### Detection of virulence and biofilm-associated genes

The PCR for detection of virulence genes used a 25 μl reaction mixture containing 12.5 μl 2×EasyTaq PCR Super MIX (TransGen Biotech Co., Ltd, Beijing, China), 2 μl DNA, 1 μl each of forward and reverse primers (10 μmol), and 8.5 μl ultrapure water. The thermal cycling program comprised an initial denaturation at 94°C for 3 min, followed by 30 cycles of denaturation at 94°C for 30 s, annealing for 30 s, and extension at 72°C for 30 s, then, a final extension at 72°C for 5 min. The annealing temperatures are listed in Table 1. The resulting products were resolved on a 1.5% agarose gel.

### Biofilm analysis

Identification of biofilm-forming strains was performed through the MtP assay as previously described (9, 32, 33). Pure staphylococcal cultures were grown in LB broth at 37°C for 24 h, adjusted to an optical density at 630 nm (OD_630_) of 1.0, and then diluted 1:100 with biofilm-inducing medium (tryptone 10 g/L, yeast extract 5.0 g/L, NaCl 2.5 g/L, KH_2_PO_4_ 3.0 g/L, K_2_HPO_4_ 7.0 g/L, (NH_4_)_2_SO_4_ 2.0 g/L, FeSO_4_ 0.5 mg/L, MgSO_4_ 1.0 g/L, and thiamine hydrochloride 2.0 g/L) (Oxoid, UK). After incubation for 24 h at 37°C, planktonic bacteria were washed out and the biofilm was stained with crystal violet (Sangon, China). Negative controls consisted of the biofilm-inducing medium only. Each strain was analyzed in triplicate on the same plate, and three independent plates were used. The absorbance (570 nm) of the negative controls was used to set the optical density cut-off (ODc) as three SDs above the mean OD of the negative control. Strains were classified as follows: not adherent OD≤ODc; weakly adherent ODc<OD≤2×ODc; and strongly adherent OD>4×ODc.

### Statistical analysis

Statistical analysis was performed with SPSS Statistics 22.0 software (IBM, USA). Chi-square tests were used to analyze the difference. A *P* value of <0.05 was deemed statistically significant.

## Results

### Frequency of bacterial isolation in healthy dogs

The isolated microorganisms and the frequency of their isolation from the healthy dogs are shown in Table 2. Among the 70 samples isolated from the 35 healthy dogs, the positive rate of bacterial isolation was 72.9% (51/70), and the negative rate was 27.1% (19/70). Among them, positive binocular dogs accounted for 62.9% (22/35), positive monocular dogs accounted for 17.1% (6/35), and negative binocular dogs accounted for 20% (7/35). A total of 67 aerobic bacterial strains were isolated, of which 86.6% (58/67) were Gram-positive and 13.4% (9/67) were Gram-negative. *Staphylococcus* spp. accounted for the largest proportion of aerobic bacterial isolates, 41.79% (28/67), of which *S. pseudintermedius* had the highest isolation rate at 20.9% (14/67). The second largest proportion was *Bacillus* spp. with 11.94% (8/67), of which *Bacillus cereus* (*B. cereus*) had the highest isolation rate at 8.14% (7/67).

**Table 2:** Bacterial species isolated from canine conjunctival sac.

**Organism**	**Clinical healthy dog**		**Clinically dogs with ulcerative keratitis**	
	**Frequency**	**%**	**Frequency**	**%**
*Staphylococcus* spp	28	41.79	39	60.00
*S. pseudintermedius*	14	20.90	15	23.08
*S. intermedius*	4	5.97	1	1.54
*S. aureus*	0	0	3	4.62
*S. saprophyticus ssp*	0	0	2	3.08
*S. lentus*	0	0	2	3.08
*S. cohnii*	0	0	2	3.08
*S. capitis*	2	2.99	3	4.62
*S. lugduensis*	1	1.49	1	1.54
*S. epidermidis*	3	4.48	2	3.08
*S. simulans*	0	0	4	6.15
*S. equorum*	2	2.99	1	1.54
*S. sciuri ssp sciuri*	1	1.49	1	1.54
*S. auricularis*	1	1.49	2	3.08
*Bacillus* spp	8	11.94	7	10.77
*B. cereus*	7	10.45	7	10.77
*B. pumilus*	1	1.49	0	0
*Lysinibacillus* spp	1	1.49	0	0
*L. fusiformis*	1	1.49	0	0
*Weissella* spp	4	5.97	1	1.54
*W.viridescens*	3	4.48	1	1.54
*W. confusa*	1	1.49	0	0
*Enterobacter* spp	6	8.96	2	3.08
*E. hirae*	2	2.99	1	1.54
*E. faecium*	2	2.99	1	1.54
*E. durans*	1	1.49	0	0
*E. cloacae*	1	1.49	0	0
*Neisseria* spp	0	0	1	1.54
*N. zoodegmatis*	0	0	1	1.54
*Pasteurella* spp	0	0	3	4.62
*P. stomatis*	0	0	3	4.62
*Moraxella_*spp	1	1.49	2	3.08
*M. canis*	1	1.49	2	3.08
*Macorcoccus* spp	3	4.48	2	3.08
*M. caseolyticus*	3	4.48	2	3.08
*Streptococcus* spp	3	4.48	1	1.54
*S.lutetiensis*	1	1.49	0	0
*S. canis*	1	1.49	0	0
*S. oralis*	1	1.49	1	1.54
*Arcanobacterium* spp	1	1.49	0	0
*A. pluranimalium*	1	1.49	0	0
*Escherichia* spp	2	2.99	1	1.54
*E. coli*	2	2.99	1	1.54
*Rothia* spp	3	4.48	2	3.08
*R. nasimurium*	3	4.48	2	3.08
*Pseudomonas* spp	1	1.49	3	4.62
*P. aeruginosa*	1	1.49	3	4.62
Unknow	6	8.96	1	1.54

### Frequency of bacterial isolation in dogs with keratitis

The isolated microorganisms and their frequency of isolation from dogs with keratitis are shown in Table 2. In total, forty dogs with keratitis were tested, and 49 samples were collected. Among them, 31 cases (77.5%) occurred in one eye, and 9 cases (22.5%) occurred in both the eyes. There were 19 cases of superficial corneal ulcer (38.8%), 14 cases of deep corneal ulcer (28.6%), 12 cases of corneal edema (24.5%), 3 cases of corneal perforation (6.12%), and 1 case of corneal vascularization (2.04%). Out of the 49 collected samples, 45 (91.84%) yielded bacterial growth, while 4 (8.16%) were negative for bacterial growth. A total of 65 aerobic bacteria were isolated from the 49 samples. Gram-positive bacteria accounted for 81.54% (53/65) of isolates, and Gram-negative bacteria accounted for 18.46% (12/65). The isolation rate of *Staphylococcus* was the highest in 49 samples, accounting for 60% (39/65) of all the isolates, and the isolation rate of *S. pseudintermedius* was 23.08% (15/65). The isolation rate of *Bacillus* ranked second with 10.77% (7/65), of which all the isolates were *B. cereus*.

The percentage of dogs with bacterial eye diseases was calculated for breeds in which five or more dogs were examined (Table 3). The highest prevalence of bacterial eye diseases was in french bulldog (20%), followed by pug (17.5%), poodle (12.5%), chihuahua (12.5%), pekingese (10%), and bulldog (10%). The isolation rate for *S. pseudintermedius* was higher (*P* < 0.05) in brachycephalic dogs (12/40, 30%) compared with other breeds (3/40, 7.5%).

**Table 3:** Frequency of bacterial eye disease among breeds in 40 dogs.

**Breeds**	**Number (%)**	**Separation rate for *S. p* (*n =* 15)**
French Bulldog	8 (20)	5/8 (62.5)
Pug	7 (17.5)	4/7 (57.1)
poodle	5 (12.5)	2/5 (40)
Chihuahua	5 (12.5)	2/5 (40)
Pekingese	4 (10)	0/4 (0)
Bulldog	4 (10)	1/4 (25)
Other	7 (1.75)	1/7 (14.3)

### Antibiotic susceptibility tests

The antibiotic resistance of all the 29 isolates of *S. pseudintermedius* is shown in Table 4. The Kirby–Bauer assay demonstrated that 24.1% isolates were MDR–*S. pseudintermedius* (Table 5), with a higher (*P* > 0.05) number of strains isolated from dogs with keratitis (17.2%) compared with the healthy dogs (6.9%). The 29 isolates of *S. pseudintermedius* exhibited marked resistance against tetracycline (96.6%) and erythromycin (79.3%). The antimicrobials that were most effective *in vitro* were neomycin (96.6% sensitive), rifampin (96.6% sensitive), tobramycin (93.1% sensitive), and oxacillin (100% sensitive). The resistance rates for erythromycin, tobramycin, levofloxacin, and chloramphenicol were higher (*P* < 0.05) in the strains isolated from dogs with keratitis (93.3, 13.3, 26.7, and 60%, respectively) compared with the strains isolated from the healthy dogs (64.3, 0, 7.1, and 14.3%, respectively).

**Table 4:** Antimicrobial resistance of the 29 isolates of *S. pseudintermedius* from canine eyes.

**Antibiotics tested**	**Zone diameter of resistance**	**Keratitis** **(*****n** =* **15)**		**Healthy** **(*****n** =* **14)**		**Total** **(*****n** =* **29)**	
		**R**	**S**	**R**	**S**	**R**	**S**
Erythromycin	≤18	14 (93.3)^a^	1 (6.7)	9 (64.3)	5 (35.7)	23 (79.3)	6 (20.1)
Rifampin	≤23	1 (6.7)	14 (93.3)	0 (0)	14 (100)	1 (3.4)	28 (96.6)
Chloramphenicol	≤18	9 (60)^a^	8 (53.3)	2 (14.3)	10 (71.4)	11 (37.9)	18 (62.1)
Tetracycline	≤19	15 (100)	0 (0)	13 (92.9)	1 (7.1)	28 (96.6)	1 (3.4)
Neomycin	≤12	1 (6.7)	14 (93.3)	0 (0)	14 (100)	1 (3.4)	28 (96.6)
Ciprofloxacin	≤15	4 (26.7)	11 (73.3)	2 (14.3)	12 (85.7)	6 (20.7)	23 (79.3)
Tobramycin	≤12	2 (13.3)^a^	13 (86.7)	0 (0)	14 (100)	2 (6.9)	27 (93.1)
Levofloxacin	≤13	4 (26.7)^a^	12 (80)	1 (7.1)	12 (85.7)	4 (13.7)	25 (86.3)
Oxacillin	≤18	0 (0)	15 (100)	0 (0)	14 (100)	0 (0)	29 (100)

“a” means p < 0.05, vs. healthy groups.

**Table 5:** The MDR patterns of 7 *S. pseudintermedius* isolates.

**MDR patterns**	**Number (%) of positive isolates**		
	**Keratitis *n =* 5**	**Healthy *n =* 2**	**Total *n =* 7**
TE-E-RA-LVX	1 (20)	0 (0)	1(14.3)
TE-E-N-TO	1 (20)	0 (0)	1(14.3)
TE-E-TO-C	1 (20)	0 (0)	1(14.3)
TE-E-LVX-CIP	2 (40)	0 (0)	2 (28.3)
TE-LVX-CIP-C	0 (0)	1 (50)	1(14.3)
TE-E-CIP-C	0 (0)	1 (50)	1(14.3)

### Virulence factors

Table 6 shows the distribution of all the virulence genes analyzed in this study. There was universal carriage of the tested genes, namely, *ebpS* (93.1%), *spsE* (72.4%), *hlgA* (75.9%), *hlgB* (96.6%), *siet* (96.6%), *lukS-I* (93.1%), *PVL* (89.7%), and *lip* (96.6%); the exceptions were *lukF-I* (0%) and *tsst* (0%). The virulence factors *spsE, hlgA, siet*, and *PVL* were detected in more (*P* < 0.05) of the strains isolated from the dogs with keratitis (93.3, 86.7, 100, and 93.3%, respectively) compared with the strains isolated from the healthy dogs (50, 64.3, 92.3, and 85.7%, respectively). Furthermore, 10 different virulence gene patterns were observed among the isolates of *S. pseudintermedius* (Table 7). The most frequent virulence pattern was no. 1 and this pattern was detected in 48.3% of the isolates. The detection rate of virulence pattern no. 1 was higher (*P* < 0.05) in strains from the dogs with keratitis (66.7%) compared with that in strains from the healthy dogs (28.3%).

**Table 6:** Distribution of virulence factors genes among 29 isolates of *S. pseudintermedius*.

**Virulence factors**	**Number (%) of positive isolates**		
	**Keratitis** ***n =* 15**	**Healthy** ***n =* 14**	**Total** ***n =* 29**
*ebpS*	14 (93.3)^a^	13 (92.3)	27 (93.1)
*spsE*	14 (93.3)^a^	7 (50)	21 (72.4)
*hlgA*	13 (86.7)^a^	9 (64.3)	22 (75.9)
*hlgB*	15 (100)	13 (92.3)	28 (96.6)
*siet*	15 (100)^a^	13 (92.3)	28 (96.6)
*lukS-I*	14 (93.3)	13 (92.3)	27 (93.1)
*lukF-I*	0 (0)	0 (0)	0 (0)
*lip*	15 (100)	13 (92.3)	28 (96.6)
*PVL*	14 (93.3)^a^	12 (85.7)	26 (89.7)
*tsst*	0 (0)	0 (0)	0 (0)
*ica A*	15 (100)	13 (92.3)	28 (96.6)
*ica D*	15 (100)	13 (92.3)	28 (96.6)
*luk I*	0 (0)	0 (0)	0 (0)
*ica A+ ica D*	15 (100)	13 (92.3)	28 (96.6)

“a” means p < 0.05, vs. healthy groups.

**Table 7:** Virulence gene patterns of 29 isolates of *S. pseudintermedius*.

**No**.	**Virulence gene patterns**	**Keratitis *n =* 15**	**Healthy *n =* 14**	**Total *n =* 29**
1	*ebpS-spsE-hlgA-hlgB-siet-lukS-I-PVL-lip-ica A-ica D*	10 (66.7)^a^	4 (28.3)	14 (48.3)
2	*ebpS-hlgB-siet-lukS-I-PVL-lip-ica A-ica D*	0 (0)	2 (14.3)	2 (6.9)
3	*ebpS-spsE-hlgB-siet-lukS-I-PVL-lip-ica A-ica D*	2 (13.3)	1 (7.1)	3 (10.3)
4	*ebpS-spsE-hlgA-hlgB-siet-lukS-I-lip-ica A-ica D*	1 (6.7)	1 (7.1)	2 (6.9)
5	*ebpS-hlgA-hlgB-siet-lukS-I-lip-ica A-ica D*	1 (6.7)	1 (7.1)	2 (6.9)
6	*ebpS-hlgA-hlgB-siet-lukS-I-PVL-lip-ica A-ica D*	0 (0)	3 (21.4)	3 (10.3)
7	*hlgA-hlgB-PVL-lip-ica A-ica D*	1 (6.7)	0 (0)	1 (3.4)
8	*ebpS-hlgA-hlgB-siet-lukS-I-PVL-ica A-ica D*	0 (0)	1 (7.1)	1 (3.4)
9	*spsE-PVL-ica A-ica D*	0 (0)	1 (7.1)	1 (3.4)
10	*ebpS-hlgA-hlgB-siet-lukS-I-PVL-lip*	0 (0)	1 (7.1)	1 (3.4)

“a” means p < 0.05, vs. healthy groups.

### Biofilm formation assay

Biofilm formation was tested in the 29 isolates of *S. pseudintermedius* and differences among the strains are shown in Table 8. Among the isolates of *S. pseudintermedius*, 10.3% were not adherent, 44.8% were weakly adherent, 10.2% were moderately adherent, and 24.1% were strongly adherent. The relationship between source and biofilm-formation ability of the isolates of *S. pseudintermedius* was further analyzed (Table 8). There was a significantly higher proportion of strong and moderate biofilm producers in the strains isolated from the dogs with keratitis (40 and 33.3%) compared with those isolated from the healthy dogs (7.1 and 7.1%, *P* < 0.05). Conversely, there was a significantly lower proportion of weak biofilm producers in the strains isolated from the dogs with keratitis (13.3%) compared with the strains isolated from the healthy dogs (78.6%, *P* < 0.05).

**Table 8:** Biofilm-forming ability of 29 strains of *S. pseudintermedius*.

**Origin**	**Number (%) of** ***S. pseudintermedius*** **with biofilm production**			
	**Not**	**Weakly**	**Moderately**	**Strongly**
Keratitis (*n =* 15)	1 (6.7)	2 (13.3)^a^	5 (33.3)^a^	6 (40)^a^
Healthy (*n =* 14)	2 (14.3)	11 (78.6)	1 (7.1)	1 (7.1)
Total (*n =* 29)	3 (10.3)	13 (44.8)	6 (10.2)	7 (24.1)

“a” means p < 0.05, vs. healthy groups.

At the molecular level, the presence of *icaA* and *icaD* genes was demonstrated by amplifying corresponding sequences. Amplicons for *icaA, icaD*, and *icaA* with *icaD* were all the detected in 96.55% of isolates of *S. pseudintermedius*. One strain was negative for both genes and was unable to produce biofilm. Two strains were positive for both the genes but were unable to produce biofilm. The details are shown in Table 6.

## Discussion and conclusion

The methods used to determine the canine conjunctival flora varies and include dry swabs, pre-moistened swabs, calibrated platinum loop, cytologic examination of exudate, and scrapings from the conjunctival sac (37, 38). Compared with the dry swabs, pre-moistened swabs were reported to be an effective sample collection technique for bacterial composition concerning the Gram-positive isolates (39). The methods for cytologic examination or scraping of the conjunctival sac usually require application of a topical anesthetic, but some reports showed that common topical anesthetics were bacteriostatic and may decrease organism yield (38). Consequently, this method could cause false diagnosis because of the bacteriostatic effect of the topical anesthetics. Therefore, in the current study, pre-moistened swabs were used for specimen collection.

The type and frequency of bacteria isolated from the canine conjunctival sac vary among investigators and locations (2, 34, 37, 40, 41). However, a common factor evident in all the research is that Gram-positive bacteria, especially, *Staphylococcus* spp., were the organisms most frequently isolated in the healthy dogs (2, 15), and the findings of the current study were consistent with this. Investigations of *S. intermedius* before 2008 showed that *S. intermedius* was the most common isolate from the conjunctival sac of the healthy dogs and the dogs with cornea ulceration (2, 6, 37). However, the isolation rate of *S. intermedius* in the conjunctival sac has decreased in the recent years (15, 41). In the current study, the isolation rate of *S. intermedius* was 4.49 and 1.57% in the corneas of the healthy dogs and the dogs with keratitis, respectively. In contrast, the isolation rate of *S. pseudintermedius* has increased in recent years (15, 42). In the present study, the isolation rate of *S. pseudintermedius* was 20.9 and 23.8% in the corneas of the healthy dogs and the dogs with keratitis, respectively, which was consistent with previous surveys (15, 42). *S. pseudintermedius* forms part of the resident flora in the conjunctival sac of the healthy dogs and those with keratitis in Yangzhou, China. *B. cereus* was also a frequently isolated organism in the healthy dogs (7.87%) and the dogs with corneal ulceration (10.77%) in our study. According to the previous literature, *B. cereus* is the second most common group of infecting organisms isolated in human cases of post-traumatic endophthalmitis (43, 44).

The conjunctival sac is warm and humid, suitable for the growth of microorganisms, which can be divided into resident flora and opportunistic pathogenic flora (2). The current study showed that there was little difference in the flora of the conjunctival sac between the healthy dogs and the dogs with keratitis in Yangzhou; all the isolated strains could be detected as the resident flora. Primary infectious keratitis in dogs is uncommon (45). Corneal infection is usually enhanced by disruption of the defense mechanism of the ocular surface, which includes the structural integrity and blinking action of the eyelids, and the intact epithelium of the conjunctiva and cornea (46). Microtrauma is the predominant factor contributing to the development of keratitis; other causes are tear film disturbances, disorders of the immune system (immunodeficiencies and autoimmune diseases), and corneal surgical interventions (2). If a corneal ulcer develops, regardless of the underlying cause, *Staphylococcus* spp. and *Bacillus* spp. are likely to invade the ocular surface of dogs.

*S. pseudintermedius* is a Gram-positive, facultatively anaerobic coccus. Colonies are medium-sized, raised, and unpigmented, and display a large incomplete β-hemolysis and small complete δ-hemolysis either alone or in combination (double hemolysis) on sheep or bovine blood agar. As a zoonotic bacterium, *S. pseudintermedius* can cause catheter-related bloodstream infections (47), sinonasal infections (48), bacterial peritonitis (49), and venous access port infections (36). As an opportunistic pathogen, *S. pseudintermedius* is associated with skin, ear, and post-surgical infections in the domestic animals, especially dogs (50–52). In the previous studies, 11 (9.7%) strains of *S. pseudintermedius* were isolated from 113 dogs with septic keratitis (4), and 27 (25.2%) strains of *S. pseudintermedius* were isolated from 56 canine eyes with ulcerative bacterial keratitis (14). In a survey from 2018 to 2020, the frequency of isolates of *S. pseudintermedius* among 155 samples from canines with ulceration keratitis was 16% (42). It is difficult to distinguish between *S. intermedius* and *S. pseudintermedius* using traditional standard biochemical tests because of the high similarity in gene sequence and characteristics of the two species. In previous reports, many pyodermas and skin infections with *S. pseudintermedius* from pet dogs were frequently misdiagnosed as *S. intermedius* (12, 53, 54). Mass spectrometry is an effective method for the diagnosis of *S. pseudintermedius*, and the accuracy has been verified by Yisong Zhu (55).

*S. pseudintermedius* is considered as a potential zoonotic agent and is currently regarded as one of the increasing skin, eye, and soft tissue-related pathogens of humans (56–58). Antibiotic resistance of *S. pseudintermedius* is a major problem in the treatment and control of human and animal infections. MDR strains are defined as “resistant to 3 or more antimicrobial categories” (35). In a previous report, 57.5% (42/73) isolates of *S. pseudintermedius* from the clinical samples were MDR strains, and a large portion of isolates showed a pattern of resistance to β-lactams, macrolides, and fluoroquinolones (9). Research on antimicrobial resistance changes for *S. pseudintermedius* from 2005 to 2013 determined that the resistance for narrow-spectrum β-lactams has increased significantly (16). In the current study, the isolates of *S. pseudintermedius* exhibited resistance to tetracycline (96.6%), erythromycin (79.3%), and chloramphenicol (37.9%), but were sensitive to the quinolones. A study in Bangladesh found that 84.7% of *S. pseudintermedius* strains were resistant to erythromycin, and 6% of isolated strains were MDR (59). In the present study, 24.1% of the isolates were defined as the MDR *S. pseudintermedius* phenotype, and there was a larger percentage in the dogs with keratitis compared with in the healthy dogs. In addition, MDR *S. pseudintermedius* carriage was reported to be common in dogs after recovery from infection, and MDR-infected dogs are more likely to be reinfected with *S. pseudintermedius* (60). Thus, treating the disease induced by MDR *S. pseudintermedius* is very challenging.

The major *Staphylococcal* spp. virulence factor is the ability to produce biofilms, thereby facilitating adherence to biotic and abiotic surfaces. Most (89.66%) of the 29 isolates of *S. pseudintermedius* in the current study were biofilm producers, and this was congruent with the previous research on biofilms in clinical isolates of *Staphylococcus* spp. (9, 28, 61). Biofilm-related infections have attracted attention because the bacteria could be more tolerant to antibiotics and more resistant to host immune responses compared with the equivalent planktonic forms (62). Antibiotics often fail to penetrate biofilms due to the extracellular matrix, which limits the transport of antimicrobial agents. The current study revealed that there was a greater proportion of strong biofilm producers among the isolates of *S. pseudintermedius* from the dogs with keratitis (40%) compared with those from the healthy dogs (7.1%). Stronger biofilm formation may help the strains isolated from dogs with keratitis to resist tear washout and the bactericidal effect of antibiotics, which in turn enhances infection and increases the difficulty of treatment. The genetic test results for *ica A* and *ica D* in the present study were inconsistent with the observed biofilm-formation ability, and also did not agree with the current literature (9). These findings suggest that the relationship between biofilm production and the presence of genes is not clear. However, this is discordant with both a previous study where biofilm formation occurred when both *ica A* and *ica D* were expressed (9) and also the reported absence of correlation between the presence of both *ica* genes and biofilm formation (*ica*-independent biofilm-producing strains) (63). These results suggest the importance of combined phenotypic and genetic methods for checking biofilm formation in *S. pseudintermedius*.

Eye infections caused by *S. pseudintermedius* must commence with efficient colonization of the animal cornea. The presence of gene-encoding extracellular matrix-binding proteins of host tissues (*ebpS* and *spsE*) and the lipase (*lip*) enhancing the colonization of the cornea, and also the gene encoding the epidermolysis toxin, and exfoliative toxin (*siet*) were common in strains of *S. pseudintermedius* (9). Data from Kmieciak and Szewczyk indicated that virulence factors such as *ebpS, spsE, lip*, and *siet* were involved in promoting adhesion and invasion in the pathogenesis of superficial infections (10). Furthermore, Terauchi et al. suggested that s*iet* was associated with corneal epidermis colonization (64). In the current study, detection rates of adhesion-related virulence genes *ebpS* and *spsE* were, respectively, 93.1 and 72.4%, and the carriage rates of invasion-related virulence genes *siet* and *lip* were, respectively, 96.6 and 96.6% among all the isolates of *S. pseudintermedius* (i.e., those from dogs with keratitis and from the healthy dogs). However, there were some differences in virulence factor carriage rate between the isolates of *S. pseudintermedius* from the healthy dogs and those from dogs with keratitis. The detection rates of adhesion (*spsE*), invasion (*siet* and *lip*), and γ-hemolysis (*hlgA*)-related virulence factors were higher in the strains isolated from dogs with keratitis (93.3, 100, 100, and 86.7%, respectively) compared with those in the isolates from the healthy dogs (50, 92.3, 92.3, and 64.3%, respectively). This indicates that the keratitis induced by *S. pseudintermedius* may be associated with fibrinogen/fibronectin-binding protein SpsE, lipase, exfoliative toxin SIET, and γ-hemolysin A component.

PVL, together with γ-hemolysin, *luk I*, and another leukocidin (e.g., bovine leukocidin R) belongs to the recently described family of synergohymenotropic toxins (65). These toxins damage membranes of host defense cells and erythrocytes by the synergistic action of two non-associated classes of secretory proteins designated *S* and *F* (31). PVL was confirmed to be associated with pneumonia, infective endocarditis, osteomyelitis, and enterocolitis (31). *Luk I*, in which the characterization and sequence analysis were similar to *PVL*, comprises of two co-transcribed genes, *lukS* and *lukF* (referred to elsewhere as *lukS-I* and *lukF-I*, respectively), encoding LukS and LukF (66). *S. pseudintermedius* with gene *PVL* was first reported in 2018 (10). In the current study, the *PVL* gene was detected in 89.7% of all the isolates of *S. pseudintermedius*, which is consistent with the previous research (66). For the *luk I* genes, 93.1% of the isolates of *S. pseudintermedius* carried *lukS-I* but none of the isolates harbored *lukF-I* genes, indicating that none of the isolates could produce *luk I*. These results for *luk I* genes were not consistent with the previous research (9, 10). The results imply that PVL was the major leukocidin in the current study.

In conclusion, *S. pseudintermedius* was the common bacterium in the conjunctivital sac of the healthy dogs and dogs with keratitis. The biofilm-formation ability and rates of virulence gene carriage and antibiotic resistance were higher in the isolates of *S. pseudintermedius* from dogs with keratitis compared with those from the healthy dogs. Moreover, as a potential zoonotic agent, more attention should be paid to the epidemiological information for *S. pseudintermedius* in the future.

## Data availability statement

The datasets presented in this study can be found in online repositories. The names of the repository/repositories and accession number(s) can be found below: https://www.ncbi.nlm.nih.gov/genbank/, SUB11157782.

## Ethics statement

The animal study was reviewed and approved by Yang Zhou University (No: 202011003). Written informed consent was obtained from the owners for the participation of their animals in this study.

## Author contributions

HW and ZW were in charge of manuscript drafting and designed the study. JuL carried out primary canine treatment. ZW and LG performed patient care and examinations. ZW, LG, JuL, JiL, LC, JD, XM, and HW supervised the study and wrote the manuscript. All authors had agreed with our eventual version of the manuscript.

## Funding

This work was financially by 333 High-level Talent Training Project of Jiangsu Province (CN), 2021 Jiangsu Postgraduate Research and Innovation Plan (KYCX21_3273), National Key R & D Program (2016YFD0501010), Priority Academic Program Development of Jiangsu Higher Education Institution (PAPD), and Top-notch Academic Programs Project of Jiangsu Higher Education Institutions (TAPP).

## Conflict of interest

The authors declare that the research was conducted in the absence of any commercial or financial relationships that could be construed as a potential conflict of interest.

## Publisher's note

All claims expressed in this article are solely those of the authors and do not necessarily represent those of their affiliated organizations, or those of the publisher, the editors and the reviewers. Any product that may be evaluated in this article, or claim that may be made by its manufacturer, is not guaranteed or endorsed by the publisher.
